# The association of social frailty with intrinsic capacity in community-dwelling older adults: a prospective cohort study

**DOI:** 10.1186/s12877-021-02466-6

**Published:** 2021-09-27

**Authors:** Chi Hsien Huang, Kiwako Okada, Eiji Matsushita, Chiharu Uno, Shosuke Satake, Beatriz Arakawa Martins, Masafumi Kuzuya

**Affiliations:** 1 Department of Community Health and Geriatrics, Nagoya University Graduate School of Medicine, 65 Tsuruma-cho, Showa-ku, Nagoya, Aichi Prefecture Japan; 2 Department of Family Medicine, E-Da Hospital, No.1, Yida Road, Jiaosu Village, Yanchao District, Kaohsiung City, 82445 Taiwan, R.O.C.; 3 School of Medicine for International Students, College of Medicine, I-Shou University, No.8, Yida Rd., Jiaosu Village, Yanchao District, Kaohsiung City, 82445 Taiwan, R.O.C.; 4 Graduate School of Nutritional Sciences, Nagoya University of Arts and Sciences, Takenoyama-57, Iwasakicho, Nisshin, Aichi Prefecture Japan; 5 Institutes of Innovation for Future Society, Nagoya University, NIC, Chikusa Ward, Furocho, Nagoya, Aichi Prefecture Japan; 6 Section of Frailty Prevention, Department of Frailty Research, Center for Gerontology and Social Science, National Center for Geriatrics and Gerontology, 7-430 Morioka-cho, Obu City, Aichi Prefecture Japan; 7 Department of Geriatric Medicine, Hospital, National Center for Geriatrics and Gerontology, 7-430 Morioka-cho, Obu City, Aichi Prefecture Japan; 8 Adelaide Geriatrics Training and Research with Aged Care (G-TRAC Centre), Discipline of Medicine, Adelaide Medical School, University of Adelaide, 61 Silkes Rd, Paradise, Adelaide City, Sa 5075 Australia; 9 National Health and Medical Research Council Centre of Research Excellence in Frailty and Healthy Ageing, University of Adelaide, The University of Adelaide, Adelaide City, Sa 5005 Australia

**Keywords:** Healthy aging, Successful aging, Cognition, Psychological function, Vitality

## Abstract

**Background:**

Social frailty is associated with poor health outcomes; however, its effects on healthy aging indicators have not been adequately investigated. This study assessed the longitudinal association between social frailty and the intrinsic capacity of community-dwelling older adults.

**Methods:**

A total of 663 participants (56.7% women) aged ≥60 years from in Nagoya, Japan, were included in the study. The first measurement occurred in 2014, and annual follow-ups occurred until 2017. Social frailty was determined based on four items: financial difficulty, household status, social activity, and regular contact with others. A deficit score of 0 represented social robustness, 1 represented social prefrailty, and ≥ 2 represented social frailty. Intrinsic capacity was evaluated by the locomotion, cognition, psychological function, vitality, and sensory function domains. The longitudinal association was analyzed using generalized estimating equations.

**Results:**

The prevalence of social prefrailty and social frailty at baseline was 31.2 and 6.3%, respectively. The social prefrailty group (β = − 0.132, *P* < 0.001) and social frailty group (β = − 0.258, *P* < 0.001) were associated with a greater reduction in the composite intrinsic capacity scores than the social robustness group, especially in the cognition, psychological function, and vitality domains. Men with social prefrailty/social frailty demonstrated a greater decrease in the psychological function domain score (− 0.512 vs. − 0.278) than women. Additionally, the cognition domain score only decreased in men in the social prefrailty/social frailty group (β = − 0.122, *P* = 0.016).

**Conclusions:**

Social frailty was associated with intrinsic capacity and its subdomains longitudinally. Men with social frailty were more vulnerable than women to a decline in their psychological function and cognition domains. Therefore, the advanced management of social frailty is necessary to facilitate healthy aging.

**Supplementary Information:**

The online version contains supplementary material available at 10.1186/s12877-021-02466-6.

## Background

In 2015, the World Health Organization (WHO) presented the novel concept of healthy aging, which it defined as “the process of developing and maintaining the functional ability that enables well-being in older age” [1]. The potential for engaging and interacting with the environment to maintain functional ability has been introduced as intrinsic capacity (IC), which is a composite of the physical and mental reserves individuals hold throughout their lives [2]. IC is constructed based on the International Classification of Functioning, Disability and Health framework, which is consistent with biological aging theory, and consists of five health-related domains: locomotion, cognition, psychological function, vitality, and sensory function [3]. This new healthy aging index potentially captures the hallmark of aging and approximates “biological” age instead of “chronological” age [4]. Nevertheless, difficulties in index construction and inconsistent selection of measures limit its use and popularity in clinical settings.

An operational definition of IC has been developed and validated to encourage public recognition and facilitate its utilization from bench to bed, using the English Longitudinal Study of Aging [2, 4]. The following direct measures have been identified for the abovementioned five domains: gait speed, five times sit-to-stand test, and static balance in the locomotion domain; verbal fluency, delayed verbal memory, and attention in the cognition domain; affect and sleep disturbance severity in the psychological function domain; hand grip, dehydroepiandrosterone, insulin-like growth factor 1, and forced expiratory volume in the vitality domain; and self-reported vision and hearing impairment in the sensory function domain [4]. This national cohort study also showed evidence of the ability of IC to predict physical functioning, either in the overall measure or in the individual domain [4]. Among nursing home residents, the locomotion and vitality domains were found to be associated with mortality and fall rates [5]. Additionally, IC was associated with age-related biomarkers such as C-reactive protein and homocysteine in community-dwelling older adults at risk of cognitive decline [6]. To prevent and reduce undesired health outcomes, exploring the potentially reversible risk factors for IC is anticipated to pave the way for intervention.

Age, sex, education, socioeconomic status, and multimorbidity are considered to influence IC [4]. Furthermore, a recent review reported that social isolation due to the lockdown during the recent dramatic COVID-19 pandemic posed a greater risk to vulnerable older adults, who could become more frail and lose their resilience for addressing these unforeseen stressors, than less vulnerable individuals [7]. The decline in physical activity following social isolation results in muscle wasting, functional dependence, cognitive decline, depression, anxiety, malnutrition, and accelerated sensory loss [7]. Therefore, there is an urgent need to explore the social impact on IC.

Social determinants of health, including structural determinants (e.g., gender, ethnicity, socioeconomic and political context), intermediary determinants (e.g., material circumstances, behaviors factors, and psychological factors), and crosscutting determinants (e.g., social cohesion and social capital), have been explored in a conceptual framework developed by WHO [8]. However, the concept is not easily adopted in clinical settings because clinical practice aims to identify deficits and abnormalities. Thus, using the concept of social frailty, combining some of the aforementioned determinants would help introduce this critical but less investigated health-related attribute into clinical practice.

Social frailty has been identified as a risk to healthy aging, which means a lack of general resources, reduced social behavior/activities, insufficient social resources, and compromised fulfillment of social needs [9]. Measures based on this model have been developed and validated in cohort studies and have been shown to predict functional impairment, physical frailty, cognitive decline, depression, and mortality among community-dwelling older adults [10–14]. In addition, a social frailty index based on a deficit-accumulation model was also reported to be associated with functional disability, physical frailty, and survival [15–17]. However, these aforementioned assessment methods requiring a great expense of time and resources have compromised their popularity and adaptability in practice. In contrast, a simple four-item social frailty screening questionnaire based on Bunt’s social frailty concept (general resources, social resources, social behavior, and the fulfillment of basic social needs) demonstrated that social frailty was associated with six-year incident disability and mortality in community-dwelling older adults [14]. Therefore, more studies are warranted to investigate the impact of social frailty on indicators of healthy aging and subsequently provide user-friendly tools to clinical practitioners.

On the other hand, although previous systematic reviews and meta-analyses have found that social isolation and loneliness were associated with cognitive function, increased risk of dementia, and psychological distress, comparisons of the impact on men and women have shown mixed results [18–20]. The population-based Helsinki Aging Study failed to find an impact of sex on the loneliness–cognition association [21]; however, one Chinese national longitudinal study reported that the impact of loneliness on cognitive function was greater in older men than older women [22]. Sex difference for the influence of social frailty on IC also need further exploration.

Based on the foregoing, we investigate the longitudinal association between social frailty and IC using a three-year cohort of community-dwelling older adults.

## Methods

### Study design and participants

We used a three-year prospective cohort study (Nagoya Longitudinal Study for Healthy Elderly), which was originally designed to monitor changes in the diet, nutrition, and oral function of community-dwelling older adults over time, to investigate the association between social frailty and IC. This cohort recruited older adults between 60 and 89 years from a community center in Nagoya, Japan from 2014 to 2017. Individuals were excluded if they had any impairments in the Barthel index of activities of daily living (e.g., eating, bathing, getting dressed, toileting, transferring, and continence), had active cancers or incurable diseases with an estimated life expectancy of 6 months or less, had severe cognitive, hearing, or visual impairment preventing them from being interviewed, and were unable to perform a 5-m walk test.

Individuals who walked with mobility aids and failed to complete the basic activities of daily living (e.g., eating, bathing, getting dressed, toileting, transferring, and continence) were excluded. The baseline characteristics and cross-sectional findings have been published elsewhere [23–26].

### Definition of social frailty

A validated four-item questionnaire derived from Bunt’s social frailty concept (general resources, social resources, social behavior, and fulfillment of basic social needs) was used to assess social frailty [9]. The questionnaire assessed the financial difficulty (need financial support vs. no need for financial support), household status (living alone vs. not living alone), social activity (non-participation in social activities vs. regular participation in social activities), and regular contact with others (total scores of the Lubben Social Network Scale: < 12 points vs. ≥12 points) [14]. As zero or one points were awarded to each component, social frailty scores ranged from 0 (i.e., socially robust) to 4 (most socially frail). These scores were recoded as social frailty (2–4 points), social prefrailty (1 point), and social robustness (0 points) [14]. In our study, the psychometric results provided evidence of convergent validity and reliability (average variance extracted = 0.50 and composite reliability = 0.65) [27].

### Definition of intrinsic capacity

As noted in the introduction, IC was constructed using five domains: locomotion, cognition, psychological function, vitality, and sensory function. Locomotion was assessed using the 5-m normal walking speed [28, 29]. Cognition was measured using a five cognitive test comprising assessment of attention, memory, visuospatial, language, and reasoning [30]. The psychological function domain was measured using the Geriatric Depression Scale-15 (GDS-15) [31]. Vitality was assessed using the Mini-Nutritional Assessment (MNA)® and hand grip strength [32, 33]. The sensory function domain was represented by self-reported hearing and visual function. For hearing, participants were asked “Do you have a problem hearing voices in your daily life (using a hearing aid if you use one), like talking on the telephone?”. For vision, participants were asked “Do you have any difficulty in seeing things close up, like the reading newspaper, even if you are wearing glasses or contact lens?” Response options were coded as with or without impairment.

Each continuous scale test was transformed into a Z-score, for which the five cognitive test was stratified by age, sex, and educational level, while hand grip strength was stratified by sex. Responses to hearing and visual function (with/without impairment) were converted into regression scores using principal component analysis [34]. The sum of the Z-scores or regression scores divided by the number of tests in each domain represented the domain Z-score or domain regression score [6]. For consistency with the other domains wherein positive scores signified better health, negative psychological function domain Z-scores were converted into positive ones and vice versa. The composite IC score, which was the main outcome measure in the analysis, was defined as the mean of the locomotion Z-score, cognition Z-score, psychological function Z-score, vitality Z-score, and sensory function regression score.

### Measures

Personal information including age, sex, educational level, socioeconomic status, body height and weight, and medical history was obtained at baseline via interviews [35]. Medical history was used to construct the Charlson Comorbidity Index (CCI). Physical activity was gauged based on the Baecke Physical Activity Questionnaire (BAQ), with a total score ranging from 5 to 15 points (the higher the level of physical activity) [36, 37].

### Statistical analyses

The baseline characteristics of the social frailty, social prefrailty, and social robustness groups were compared using a linear trend estimation in a general linear model for the continuous variables and a chi-squared test for the categorical variables. A generalized estimating equation modeled as a generalized linear model was used to investigate the longitudinal association over a three-year period. The procedure introduced by Liang and Zeger produces valid inferences when data are missing at random [38]. The estimation of the longitudinal association among the social frailty status at each time point and change in the composite and subdomain IC scores was adjusted for the sociodemographic variables (age, sex, and educational level) and health-related covariates (body mass index [BMI], CCI, and BAQ scores). Household status, which is a diagnostic criterion of social frailty, was not included in the adjustment. The β estimate, referred to as a standardized regression coefficient, was used to present the change in the variables of interest. To manage missing data, multiple imputation was performed, which allowed individuals with incomplete data to be included in the analyses [39]. We created five imputed datasets by replacing all the missing values across all the time points with a standard fully conditional specification. The parameter estimates of interest were pooled from these five imputed datasets based on Rubin’s rules [40]. All tests for significance were two-sided at the 95% confidence interval (*P* < 0.05). Statistical analyses were performed using SPSS Statistics for Windows, Version 27.0 (IBM Corp, Armonk, NY).

## Results

### Baseline characteristics

**Fig. 1:**
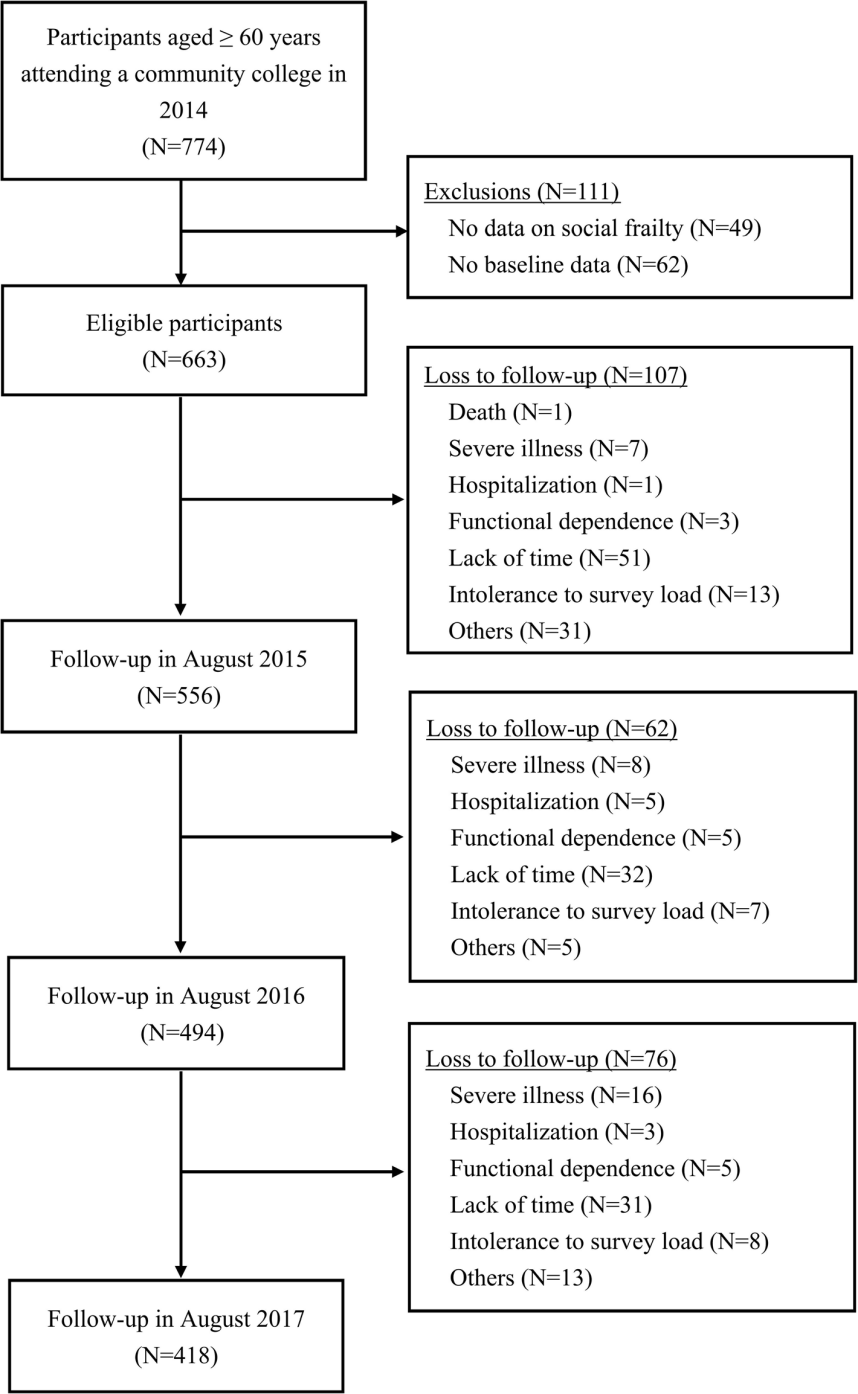
Flowchart of study participants and follow-up

At the beginning of the study, 774 older adults were invited to participate; subsequently, 49 and 62 enrollees were excluded because of incomplete information about social frailty and other baseline profile, respectively. Therefore, 663 participants were assessed annually from 2014 to 2017. A total of 107, 62, and 76 enrollees were lost to follow-up at the visits in 2015, 2016, and 2017, respectively. Figure 1 shows the details of the recruitment process, reasons, and number of dropouts. At baseline, 663 eligible participants (56.7% women) had a mean age of 69.5 (standard deviation [SD] = 4.5) years, with 89.6% aged 65 years or more. A total of 207 (31.2%) and 42 (6.3%) participants were categorized as having social prefrailty and social frailty, respectively (Table 1).

**Table 1:** Participants’ baseline characteristics by social frailty status

Variable^a,b^	Total (*N* = 663)	Social robustness (*N* = 414)	Social prefrailty (*N* = 207)	Social frailty (*N* = 42)	*P* value^*^
Age, years	69.5 (4.5)	69.3 (4.5)	69.9 (4.5)	68.9 (4.1)	0.65
Sex, N (%)					
Men	287 (43.3)	192 (46.4)	91 (44.0)	4 (9.5)	< 0.01
Women	376 (56.7)	222 (53.6)	116 (56.0)	38 (90.5)	
Educational level, N (%)					
≤ 9 years	38 (5.7)	19 (4.6)	13 (6.3)	6 (14.3)	0.01
10–12 years	305 (46.0)	178 (43.0)	107 (51.7)	20 (47.6)	
> 12 years	320 (48.3)	217 (52.4)	87 (42.0)	16 (38.1)	
Economic status, N (%)					
Need support	11 (1.7)	0 (0)	4 (1.9)	7 (16.7)	< 0.01
Self-supporting	538 (81.1)	335 (80.9)	174 (84.1)	29 (69.0)	
Well off	114 (17.2)	79 (19.1)	29 (14.0)	6 (14.3)	
BMI, kg/m^2^	22.6 (2.8)	22.6 (2.7)	22.4 (2.7)	21.7 (2.4)	0.03
CCI, scores	3.2 (1.2)	3.2 (1.2)	3.3 (1.2)	3.1 (1.2)	0.49
MNA, scores	26.1 (2.3)	26.3 (2.2)	25.7 (2.3)	25.1 (2.2)	< 0.01
GDS-15, scores	2.2 (2.7)	1.6 (2.0)	3.1 (3.3)	4.1 (3.2)	< 0.01
Physical activity (BAQ), scores	7.6 (1.3)	7.8 (1.2)	7.4 (1.3)	6.6 (1.2)	< 0.01
Usual walking speed, m/s	1.4 (0.2)	1.4 (0.2)	1.4 (0.2)	1.4 (0.2)	0.80
Composite IC score	0.1 (0.4)	0.1 (0.4)	−0.1 (0.5)	− 0.2 (0.4)	< 0.01
Locomotion domain score	0.1 (1.0)	0.1 (0.9)	−0.1 (1.0)	0.1 (1.0)	0.80
Cognition domain score	0.2 (0.7)	0.2 (0.7)	0.2 (0.7)	0.3 (0.8)	0.43
Psychological function domain score	−0.1 (1.0)	0.2 (0.8)	−0.3 (1.2)	− 0.7 (1.2)	< 0.01
Vitality domain score	−0.1 (1.0)	0.1 (0.8)	−0.1 (0.8)	− 0.5 (0.6)	< 0.01
Sensory function domain score	0.1 (1.0)	−0.1 (1.0)	0.1 (1.1)	−0.1 (0.9)	0.98

*Note*. *BMI* Body mass index, *CCI* Charlson Comorbidity Index, *MNA* Mini-Nutritional Assessment, *GDS-15* Geriatric Depression Scale-15, *BAQ* Baecke Physical Activity Questionnaire, *IC* Intrinsic capacity

^*^Significant differences (*P* < 0.05) between the social robustness group, social prefrailty group, and social frailty group were analyzed using the chi-squared test of independence for the categorical variables and a linear trend estimation in a general linear model for the continuous variables

^a^All values are mean (standard deviation) unless specified

^b^Variance inflation factor of each variable was all lower than 1.4

The demographic and baseline characteristics of the participants according to their social frailty level, as shown in Table 1, revealed that women, as well as participants with a low education, low economic status, low BMI, low MNA score, high GDS-15 score, and low BAQ score, were more vulnerable to social frailty. However, no significant differences were observed in age, CCI, and usual walking speed. The mean ± SD composite IC score for all the participants was 0.1 ± 0.4, with scores of 0.1 ± 1.0, 0.2 ± 0.7, − 0.1 ± 1.0, − 0.1 ± 1.0, and 0.1 ± 1.0 for the locomotion, cognition, psychological function, vitality, and sensory function domains, respectively. Participants with social robustness had a higher composite IC score (mean = 0.1, SD = 0.4) than those with social prefrailty (mean = − 0.1, SD = 0.5) and social frailty (mean = − 0.2, SD = 0.4). In total, 232 participants dropped out mainly because they didn’t have the time to complete the follow-up appointments during the three-year follow-up period (Fig. 1). No significant differences were observed across the baseline profiles between dropouts and non-dropouts (Additional file 1: Appendix 1).

### Impact of social frailty on intrinsic capacity

For all the participants, the composite IC scores at the end of the first, second, and third years compared with baseline were improved by 0.03 (*P* = 0.04), 0.06 (*P* < 0.01), and 0.08 (*P* < 0.01), respectively. For the participants in the social robustness group, the changes in the composite IC score were significant in all 3 years (first year: 0.04 [*P* = 0.03], second year: 0.07 [*P* < 0.01], third year: 0.12 [*P* < 0.01]). However, no significant longitudinal changes were detected for the participants in the social prefrailty and frailty groups.

After adjusting for age, sex, educational level, BMI, CCI, and physical activity level, social frailty status was associated with the composite IC scores (Table 2). These composite IC scores were lower in the social prefrailty group (β = − 0.132, *P <* 0.001) and social frailty group (β = − 0.258, *P <* 0.001) than in the social robustness group (Table 2). There were negligible differences in the sensitivity analysis findings for both participants with complete IC data and those with replaced missing values (Additional file 1: Appendix 2a and b).

**Table 2:** Longitudinal association between intrinsic capacity and associated factors^a^

Variable	GEE β estimates^b,c^	*P* value	95% CI	
			Lower limit	Upper limit
Age, years	0.002	0.612	−0.004	0.008
Sex				
Women	**−0.194**	< 0.001	−0.250	− 0.138
Men	Reference			
Educational level				
≤ 9 years	Reference			
10–12 years	**−0.162**	0.004	− 0.271	− 0.052
> 12 years	**− 0.209**	< 0.001	− 0.321	− 0.096
BMI	**0.016**	0.001	0.007	0.026
CCI	**−0.028**	0.001	−0.044	−0.012
Physical activity (BAQ), scores	**0.039**	< 0.001	0.025	0.052
Social frailty status				
Social robustness	Reference			
Social prefrailty	**−0.132**	< 0.001	− 0.175	− 0.089
Social frailty	**− 0.258**	< 0.001	− 0.357	− 0.159

*Note*. *BMI* Body mass index, *CCI* Charlson Comorbidity Index, *BAQ* Baecke Physical Activity Questionnaire

^a^Adjusted for age, sex, educational level, BMI, CCI score, BAQ score, and social frailty status

^b^GEE β estimates reflect the mean differences in the composite intrinsic capacity scores

^c^Bold values denote statistical significance at the 0.05 level

By domain, participants with social prefrailty were associated with lower composite IC scores in the cognition domain (β = − 0.081, *P* < 0.015), psychological function domain (β = − 0.322, *P* < 0.001), and vitality domain (β = − 0.138, *P* < 0.001) (Table 3). In addition, participants with social frailty were associated with lower composite IC scores in the psychological function domain (β = − 0.666, *P* < 0.001) and vitality domain (β = − 0.127, *P* = 0.021) (Table 3). However, no significant associations between social frailty and the locomotion and sensory function domains were observed.

**Table 3:** Longitudinal association between intrinsic capacity scores in each domain and social frailty status^a^

IC domain	GEE β estimates^b^	*P* value	95% CI	
			Lower limit	Upper limit
Locomotion domain score				
Social robustness	Reference			
Social prefrailty	− 0.081	0.121	− 0.184	0.021
Social frailty	−0.143	0.175	−0.351	0.064
Cognition domain score				
Social robustness	Reference			
Social prefrailty	**−0.081**	0.015	−0.146	− 0.016
Social frailty	−0.113	0.104	−0.249	0.023
Psychological function domain score				
Social robustness	Reference			
Social prefrailty	**−0.322**	< 0.001	− 0.425	− 0.220
Social frailty	**−0.666**	< 0.001	− 0.930	−0.402
Vitality domain score				
Social robustness	Reference			
Social prefrailty	**−0.138**	< 0.001	− 0.194	− 0.081
Social frailty	**− 0.127**	0.021	− 0.235	− 0.019
Sensory function domain score				
Social robustness	Reference			
Social prefrailty	0.034	0.550	−0.077	0.145
Social frailty	−0.091	0.357	−0.286	0.103

^a^Adjusted for age, sex, educational level, BMI, CCI score, and BAQ score

^b^Bold values denote statistical significance at the 0.05 level

Based on the significant IC score differences by sex, a stratified analysis by sex was performed (Table 4). The social prefrailty and social frailty groups were combined for this analysis because of the few cases in the latter group. Although no sex difference was apparent between the social prefrailty/social frailty group and social robustness group in the composite IC score (− 0.157 vs. − 0.137) and vitality domain score (− 0.130 vs. − 0.140), the psychological function domain score demonstrated a greater decrease in social prefrailty/social frailty in men than women (− 0.512 vs. − 0.278). Additionally, the cognition domain score only decreased for men with social prefrailty/social frailty (β = − 0.122, *P* = 0.016).

**Table 4:** Longitudinal association between intrinsic capacity scores and social frailty status by sex^a^

IC domain	Men				Women			
	GEE β estimates^b^	*P* value	95% CI		GEE β estimates^b^	*P* value	95% CI	
			Lower limit	Upper limit			Lower limit	Upper limit
Composite IC score								
Social robustness	Reference				Reference			
Social prefrailty or frailty	**−0.157**	**< 0.001**	**−0.219**	**− 0.094**	**−0.137**	**< 0.001**	**− 0.195**	**− 0.080**
Locomotion domain score								
Social robustness	Reference				Reference			
Social prefrailty or frailty	−0.066	0.428	−0.229	0.097	−0.112	0.087	−0.240	0.016
Cognitive domain score								
Social robustness	Reference				Reference			
Social prefrailty or frailty	**−0.122**	**0.016**	**−0.221**	**− 0.023**	−0.060	0.158	−0.144	0.023
Psychological function domain score								
Social robustness	Reference				Reference			
Social prefrailty or frailty	**−0.512**	**< 0.001**	**− 0.683**	**− 0.342**	**− 0.278**	**< 0.001**	**− 0.407**	**−0.149**
Vitality domain score								
Social robustness	Reference				Reference			
Social prefrailty or frailty	**−0.130**	**0.003**	**− 0.216**	**−0.044**	**− 0.140**	**< 0.001**	**− 0.213**	**−0.067**
Sensory function domain score								
Social robustness	Reference				Reference			
Social prefrailty or frailty	−0.029	0.736	−0.196	0.738	0.081	0.221	−0.049	0.210

^a^Adjusted for age, educational level, BMI, CCI score, and BAQ score

^b^Bold values denote statistical significance at the 0.05 level

## Discussion

Our findings indicate that social frailty is associated with IC in community-dwelling older adults. Cognition, psychological function, and vitality are the domains most susceptible to the impact of social frailty.

Based on our findings, social prefrailty and social frailty affected IC during the three-year observation period after adjusting for age, sex, education, BMI, comorbidity, and physical activity, where the latter had a stronger impact than the former. Social distancing and social segregation may result in a lack of resources and loss of support from friends, neighbors, and family, leading to lower physical and mental capability to face ensuing clinical and environmental stressors throughout life [7]. Although digital social media can expedite personal interactions and contactless communication, most older adults are excluded from social networks and online connectivity because they cannot easily access the Internet [41]. In a randomized controlled trial, a technology-based intervention strategy for social isolation was found to enhance social engagement and reduce loneliness among older adults [42]. Moreover, e-interventions may increase computer self-efficacy, proficiency, and comfort with technology, paving the way for virtual interventions in the future [43]. Hence, despite weak evidence and inconsistent findings in the literature, effective and practical interventions for social frailty need to be investigated and integrated into the healthcare model.

Consistent with previous findings, social frailty was associated with deficits in the cognition, psychological function, and vitality domains [11, 12, 44]. Social isolation accompanying loneliness triggers depressive symptoms, subjective memory decline, and the onset of dementia [11, 44]. In addition, the lack of stimulus from oral conversation and verbal communication reduces the cross-talk between the mouth and brain, further resulting in deteriorated oral function (e.g., impaired chewing ability, salivation, and tongue pressure), loss of appetite, worsening depressive symptoms, and progressive cognitive complaints [45–47]. Subsequently, malnutrition occurs, which accelerates the vicious cycle of disability and mortality [11]. By contrast, the locomotion domain represented by gait speed and the sensory function domain represented by hearing and visual ability were relatively preserved in our findings. As the major critical capabilities to maintain activities of daily life, walking, vision, and hearing may be relatively unaffected during the initial years of social isolation. Extensive follow-up with objective measurements of visual acuity and auditory function is thus necessary to reveal changes in IC over time.

This study demonstrated a difference in cognitive decline and psychological burden for older men and women with social prefrailty or frailty. Our findings corroborated that sex could play an important role in cognitive decline and psychological distress among older adults with or without social frailty. Although women reported loneliness more frequently than men, the severity of loneliness was greater in men due to the paucity of social networks and weak emotional and social support [48]. Moreover, men who felt lonely were more vulnerable to depression, low life satisfaction, and low resilience than women [49]. Even if current social promotion programs are not designed for women, most participants and potential candidates are women. Therefore, developing social promotion campaigns oriented toward older men may help improve the scores of older adults in the cognitive and psychological function domains.

Interventions aimed at managing social connectedness have been widely advocated through one-to-one personal contact, productive engagement, and group activity [50]. However, the weak and heterogeneous evidence from interventions has resulted in inconclusive benefits for social loneliness, social interactions, and social networks [51]. On the contrary, due to the concurrence of multiple comorbidities, polypharmacy, functional decline, and sensory deprivation in older adults, comprehensive assessments beyond social perspectives such as IC consisting of physical, psychological, and functional evaluation are encouraged. Furthermore, using a quantitative approach of IC measurement to investigate its association with social frailty and influence of social engagement could advance current knowledge, which is predominantly derived from qualitative research [51]. Although Beard et al. have developed a comprehensive assessing approach for IC using structural equation modeling, we tried to introduce a more intuitive and understandable analytical method for clinicians and community practitioners, which was modified from previous studies [6, 34]. However, a standardized scoring system and unanimity on variable selection would facilitate further investigation.

Our study is the first to explore the longitudinal association between social frailty and IC. The strength of this study is that it uses a prospective cohort design, although residual cofounders might still be present. It also has several limitations. First, the recruited participants from the community college were relatively young, healthy, and highly educated. The composite IC scores were improved in the social robustness group, though those scores were not changed in the social prefrailty or social frailty group at the annual follow-ups. This indicates that the findings are not applicable to unhealthy older adults who are housebound, including those living in nursing homes and those requiring assistance when going out. Since these housebound older adults are assumed to be more disabled physically and psychologically than individuals that can leave home, the severity of the impact of social frailty on their health and well-being deserves further consideration and examination. Second, our sensory function evaluation was based on the subjective reporting of visual and hearing impairment, which may lead to reporting bias. Objective measures such as visual acuity and pure tone audiometry tests are encouraged in future studies. Third, using weight for composite IC score calculation may reflect an actual status of healthy aging; however, there is no consensus about the weighted scores so far. A large-scale national cohort study could be of help to shed light on weighing the individual IC domain. Fourth, psychometric results of the social frailty scale only demonstrated modest validity and reliability, which indicate that a more accurate and reliable instrument is warranted. In addition, a multi-level approach with measurements at broader levels including intermediary determinants of health, such as material circumstances (housing and neighborhood quality, and the physical work environment) and psychosocial circumstances (psychosocial stressors, stressful living circumstances, and relationships, and social support and coping styles) would be helpful for illustrating social frailty [8]. Fifth, the high rate of attrition during follow-up diminished the credibility of the results. However, consistent findings were obtained using the multiple imputation method to replace missing values. Further investigation of the longitudinal relationships between social frailty and metrics for good health, including quality of life, comprehensive well-being, and sustainability of positive health outcomes, would be instructive and constructive for the development of public health strategies.

## Conclusions

The study revealed that social frailty was associated with IC, particularly in the cognition, psychological function, and vitality domains. Men with social frailty were more vulnerable than women to a decline in their psychological function and cognition domains. The management of social frailty should be incorporated into multifaceted prevention strategies to enable healthy aging.

## Supplementary Information

**Additional file 1.**

## Data Availability

The datasets used and/or analyzed during the current study are available from the corresponding author on reasonable request.

## Abbreviations

IC: Intrinsic capacity
GDS-15: Geriatric Depression Scale-15
MNA: Mini-Nutritional Assessment
CCI: Charlson Comorbidity Index
BAQ: Baecke Physical Activity Questionnaire
BMI: Body mass index
SD: Standard deviation
