# Supplementary material for: The association of social frailty with intrinsic capacity in community-dwelling older adults: a prospective cohort study
Source: BMC Geriatr. 2021 Sep 27;21:515. doi: 10.1186/s12877-021-02466-6 (PMC8475329; doi:10.1186/s12877-021-02466-6)
Supplement: Supplementary file 1 — Additional file 1. [file 12877_2021_2466_MOESM1_ESM.docx]

Appendix 1. Baseline characteristics by non-dropouts and dropouts

| Variable^§^ | Non-dropouts (N=431) | Dropouts (N=232) | *p* value |
| --- | --- | --- | --- |
| Age, years | 69.4 (4.4) | 69.6 (4.7) | 0.60 |
| Sex, men, N (%) | 151 (41.8) | 153 (43.6) | 0.64 |
| Educational level, N (%) |  |  |  |
| ≤9 years | 14 (4.0) | 25 (7.6) | 0.08 |
| 10–12 years | 169 (48.8) | 142 (43.4) |  |
| >12 years | 163 (47.1) | 160 (48.9) |  |
| Economic status, N (%) |  |  |  |
| Need support | 7 (2.0) | 5 (1.5) | 0.85 |
| Self-supporting | 282 (81.5) | 265 (81.0) |  |
| Well off | 57 (16.5) | 57 (17.4) |  |
| BMI, kg/m^2^ | 22.4 (2.5) | 22.8 (2.9) | 0.08 |
| CCI, scores | 3.2 (1.2) | 3.3 (1.2) | 0.50 |
| MNA, scores | 26.0 (2.3) | 26.1 (2.3) | 0.79 |
| GDS-15, scores | 2.2 (2.7) | 2.3 (2.7) | 0.58 |
| Physical activity (BAQ), scores | 7.6 (1.2) | 7.6 (1.3) | 0.62 |
| Usual walking speed, m/s | 1.4 (0.2) | 1.4 (0.2) | 0.79 |

^§^All values are mean (SD) unless specified

^#^Adjusted by actual body weight

BMI, body weight index; MNA, mini-nutritional assessment; CCI, charlson comorbidity index; GDS, geriatric depression scale; BAQ, Baecke Physical Activity Questionnaire

Appendix 2. Sensitivity analysis results for change of intrinsic capacity in (a) participants with complete intrinsic capacity data in 5 domains (b) participants with missing values of intrinsic capacity replaced

(a)

|  | All complete cases^††^ | | | |
| --- | --- | --- | --- | --- |
| Social frailty status | GEE β estimates^†*^ | P value | 95% CI | |
|  |  |  | Lower limit | Upper limit |
| Social robustness | Reference |  |  |  |
| Social prefrailty | **-0.136** | <0.001 | -0.179 | -0.093 |
| Social frailty | **-0.253** | <0.001 | -0.353 | -0.153 |

(b)

|  | Missing values replacement by multiple imputation^††^ | | | |
| --- | --- | --- | --- | --- |
| Social frailty status | GEE β estimates^†*^ | P value | 95% CI | |
|  |  |  | Lower limit | Upper limit |
| Social robustness | Reference |  |  |  |
| Social prefrailty | **-0.129** | <0.001 | -0.175 | -0.083 |
| Social frailty | **-0.244** | <0.001 | -0.339 | -0.149 |

^*^Bold values denote statistical significance at the P value < 0.05 level.

^†^GEE β estimates reflect the annual mean changes in composite intrinsic capacity scores

^††^Adjusted for age, sex, educational level, BMI, CCI score, and BAQ score
